# Supplementary material for: Comparative Analysis of Pretreatment Methods for Fruit Waste Valorization in Euglena gracilis Cultivation: Impacts on Biomass, β-1,3-Glucan Production, and Photosynthetic Efficiency
Source: Foods. 2024 Oct 28;13(21):3439. doi: 10.3390/foods13213439 (PMC11545038; doi:10.3390/foods13213439)
Supplement: Supplementary file 1 [file foods-13-03439-s001.zip › foods-3262766-supplementary.pdf]

Supplementary File

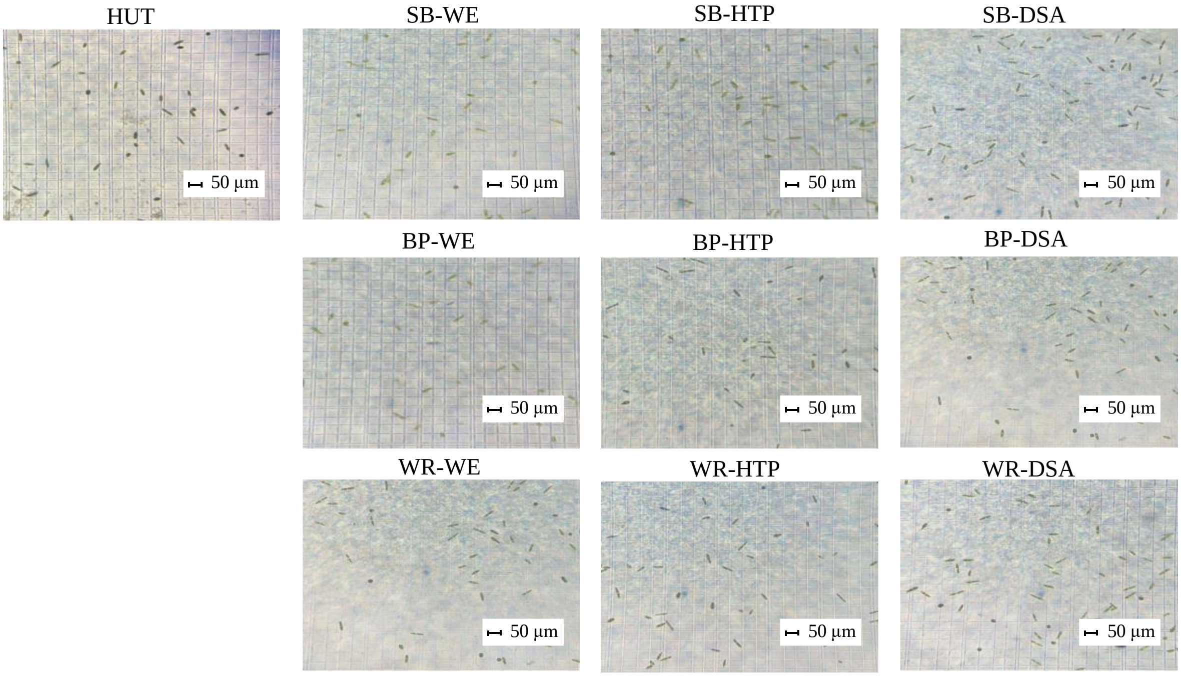

Figure S1 Optical microscope images of the morphology of *E. gracilis* cells (day 14) under different treatments. Note: At least 100 cells were randomly selected to determine AR and PA. Only a subset of cells is depicted in this image.
